# Evaluation of signal disturbance and recovery in phased array ultrasonic inspection during welding

**DOI:** 10.1007/s40194-025-02256-3

**Published:** 2025-11-13

**Authors:** Angelos Dimakos, Nina E. Sweeney, Charalampos Loukas, Christopher D. Thompson, Sam Serjeant, Charles N. MacLeod, Ehsan Mohseni, David Lines, James Sibson

**Affiliations:** 1 Centre for Ultrasonic Engineering (CUE), Department of Electronic & Electrical Engineering, University of Strathclyde, Glasgow, G1 1XQ UK; 2 Peak NDT Ltd, Jubilee Business Park, 1 Enterprise Way, Derby, DE21 4BB UK; 3 Babcock International Group, Babcock Technology Centre, Unit 100B Bristol Business Park, Stoke Gifford, Bristol, BS16 1EJ UK

**Keywords:** Ultrasonic imaging, Lack of sidewall fusion (LOSWF), In-process weld inspection, Automated welding, Artificial defect sensitivity

## Abstract

Lack of sidewall fusion (LOSWF) is a critical defect in arc welding that compromises structural integrity, especially in multi-pass welds where buried discontinuities require highly advanced volumetric imaging techniques for detection. Traditional non-destructive testing (NDT) methods are often unable to identify such defects until fabrication is complete, increasing rework rates and overall build time. This study presents a novel approach, combining in-process ultrasonic imaging with controlled experimentation to enable LOSWF detection capability during welding. An experimental setup is introduced in which a static phased array probe is positioned ahead of the welding torch, allowing B-scan acquisition in real-time, during welding. Characteristic signal loss is observed prior to sidewall fusion, followed by echo recovery upon solidification—providing a dynamic indicator of fusion status, with a distinct amplitude drop from 60 to 0%, highlighting the binary nature of the monitoring. To benchmark detection limits, artificial LOSWF flaws were introduced into single-layer welds and evaluated using a roller probe configuration. In addition, experiments were performed to analyze signal degradation and recovery due to thermal disturbance, captured through C-scan sidewall echo analysis. The results demonstrate that ultrasonic imaging deployed during welding can offer both predictive and confirmatory information about fusion quality. This integrated approach provides a foundation for automated, embedded weld inspection systems that can identify fusion defects earlier in the process chain.

## Introduction

The evolution toward intelligent manufacturing systems has positioned welding automation as a cornerstone of Industry 4.0 transformation, where real-time quality assurance becomes essential for autonomous production environments. Modern manufacturing demands have driven the integration of cyber-physical systems that bridge traditional welding processes with digital intelligence, enabling immediate process adjustments based on quality feedback. However, the transition from post-process inspection to in-process monitoring presents significant technical challenges, particularly for detecting critical defects that compromise structural integrity during fabrication.

Welding remains a fundamental joining technique in the fabrication of large-scale infrastructure such as pipelines, pressure vessels, offshore platforms, and modular structural assemblies. The long-term reliability and load-bearing capacity of these structures hinge on the integrity of their welded joints. Among the various defect modes encountered in multi-pass welding, lack of sidewall fusion (LOSWF) represents one of the most critical quality concerns due to its potential to weaken interlayer bonding, resulting in premature failure under cyclic loading or stress corrosion conditions [1, 2]. LOSWF typically manifests in narrow-groove geometries where accessibility is restricted, and poor arc control or surface contamination may cause incomplete melting of the sidewall [3]. The economic impact of undetected LOSWF is substantial—with welding representing up to 40% of total fabrication costs and defect-related repairs costing 2–3 times the original fabrication expense; there is an urgent industrial need for real-time quality assurance systems [4, 5].

Despite decades of advancement in non-destructive testing (NDT), traditional inspection methods such as visual testing (VT), and radiographic testing (RT) are inherently limited in their ability to detect interpass and subsurface defects like LOSWF during active welding. Visual inspection remains surface-dependent and incapable of identifying flaws buried within multilayer welds [6, 7]. Radiographic techniques, although suitable for planar flaw detection, struggle with fusion-type defects due to their limited volumetric sensitivity and orientation dependence [8]. These limitations create a critical gap between manufacturing requirements and quality assurance capabilities, often delaying defect discovery until post-weld inspection when repair costs escalate dramatically [9].

The convergence of intelligent manufacturing paradigms with advanced sensing technologies has created unprecedented opportunities for embedded quality assurance systems. For multi-pass welding operations, in-process detection of interpass flaws like LOSWF becomes particularly critical before subsequent layers obscure the defects [10, 11]. Automated and robotic welding systems further necessitate embedded inspection methods that provide timely feedback to prevent defect propagation and enable adaptive process control [12]. Phased array ultrasonics offers unique advantages for this application through its volumetric imaging capability, electronic beam steering, and high spatial resolution [13]. However, the deployment of ultrasonic imaging for real-time weld monitoring remains technically challenging due to thermal gradients, electromagnetic interference, and probe coupling instability in high-temperature welding environments [14, 15].

Recent research efforts have focused on advancing process-integrated NDT through controlled creation and detection of artificial defects to evaluate phased array system sensitivity under realistic welding conditions. These artificial flaws are commonly embedded within calibration blocks or fabricated in mock-up welds to simulate interpass conditions and benchmark detectability thresholds [16, 17]. Such experimental approaches enable systematic evaluation of probe configurations and acoustic response characteristics under known defect scenarios [18, 19]. Parallel investigations explore the effects of probe positioning, wedge design, and thermal interference on signal integrity during live welding, often utilizing B-scan imaging to visualize signal damping and recovery behavior [20, 21].

Phased array imaging utilizes multiple transducer elements that can be excited in programmed sequences (focal laws) to synthesize ultrasonic beams. Unlike conventional single-element probes, phased arrays can perform linear scans, where the active aperture is electronically stepped across the array, enabling coverage of a large inspection surface without mechanical probe movement. In weld fusion inspection, this approach is particularly effective when the expected defect location and orientation are known a priori [22]. In this study, a 45° linear scan was employed to insonify the sidewall region with reduced gain requirements, ensuring reliable detection of lack-of-sidewall-fusion defects while maintaining sensitivity under high-temperature welding conditions.

Recent studies (2024–2025) have advanced multi-sensor frameworks for real-time weld quality monitoring by integrating vision, thermal, acoustic, and electrical sensing with deep learning and attention-based models. For instance, He et al. [23] implemented an open-source, multi-robot directed energy deposition framework incorporating in-process thermal imaging, laser scanning, acoustic, and electrical sensors for adaptive process control and post-printing metrology. While ultrasonic testing was referenced for geometry evaluation and non-destructive testing, it remained confined to post-pass inspection rather than arc-on sensing. Xu et al. [24] further analyzed attention-based sensor fusion architectures, showing how channel and spatial attention improve feature weighting across visual, acoustic, and arc-signal domains for penetration estimation and defect prediction. Complementing these, Singh and Vasudev [25] reviewed artificial intelligence-driven sensor fusion strategies combining, vision, infrared, and acoustic data streams, highlighting challenges in robustness, real-time implementation, and domain generalization for intelligent welding systems. Beyond welding, Mohamed et al. [26] demonstrated ultrasonic-driven adaptive control in robotic plasma cutting, where real-time ultrasonic thickness sensing informed bevel geometry and cutting parameters. However, the sensing occurred prior to or between cuts rather than during an active fusion process, distinguishing it from true arc-on ultrasonic monitoring. Despite these advances, subsurface defect detection remains limited to inspection stages removed from the area of dynamic welding conditions. Ultrasonic testing continues to be applied post-pass, inter-pass, and during welding for solidified material, with notable exceptions [21]. This absence underscores a critical research gap—the integration of ultrasonic imaging into arc-on approaches for real-time detection of LOSWF and similar fusion defects.

This study presents a novel integrated approach combining real-time phased array ultrasonic monitoring with systematic artificial defect validation to advance in-process LOSWF detection capabilities. First, we demonstrate an innovative monitoring configuration where a static wedge probe positioned ahead of the welding torch enables real-time B-scan visualization of sidewall fusion during active welding. This approach captures transient signal loss and recovery patterns that correlate directly with weld pool interference and material solidification, providing an immediate proxy for fusion quality assessment. Second, we conduct controlled sensitivity studies using artificially introduced LOSWF defects in conjunction with a robotic RollerProbe inspection to establish detection thresholds and validate signal interpretation under realistic multi-pass geometries. The integration of predictive real-time monitoring with confirmatory post-process validation represents a significant advancement toward fully autonomous welding quality assurance systems. This is the first systematic integration of real-time interpass PAUT monitoring with artificial LOSWF calibration protocols, establishing foundational capabilities for embedded inspection in intelligent welding manufacturing workflows.

## Experimental setup

**Fig. 1:**
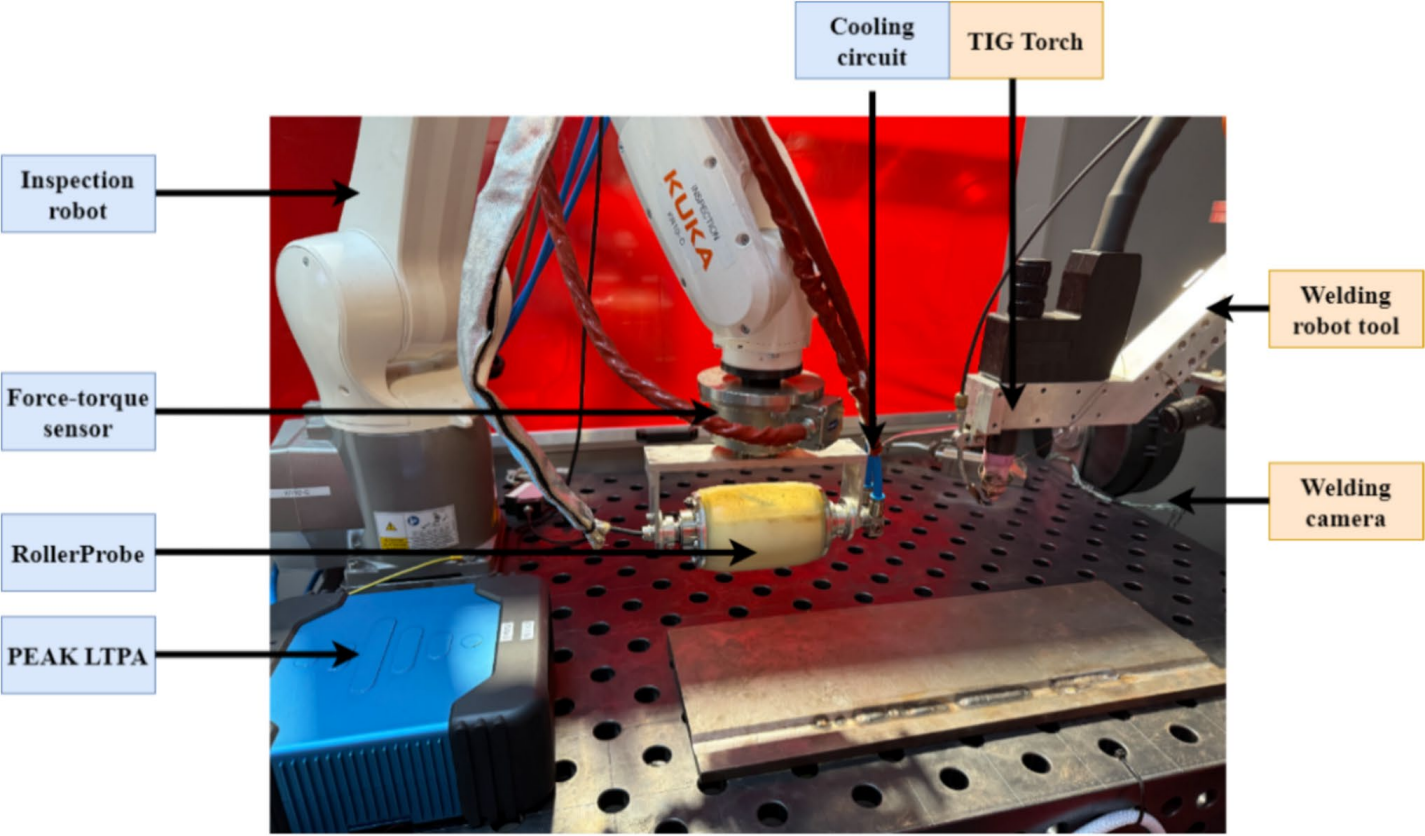
Tandem robotic setup used for welding and in-process inspection. The system includes a TIG welding robot and an inspection robot equipped with a PAUT roller probe, force–torque sensor, and PEAK LTPA system. A visual camera mounted adjacent to the weld line records arc behavior and process disturbances

The experimental system was composed of two collaborative robotic arms. One robot executed gas tungsten arc welding (GTAW), while the other carried a phased array ultrasonic RollerProbe integrated with a PEAK LTPA data acquisition unit. The RollerProbe assembly was equipped with a force–torque sensor and a water-cooling loop to maintain constant coupling and thermal stability during operation. As shown in Fig. 1, the welding robot was fitted with a GTAW torch, a wire feeder, and a side-mounted high dynamic range (HDR) visual camera to monitor the arc region. S275 structural steel was used for both the base and filler materials throughout all trials.

### Welding parameter trials

**Fig. 2:**
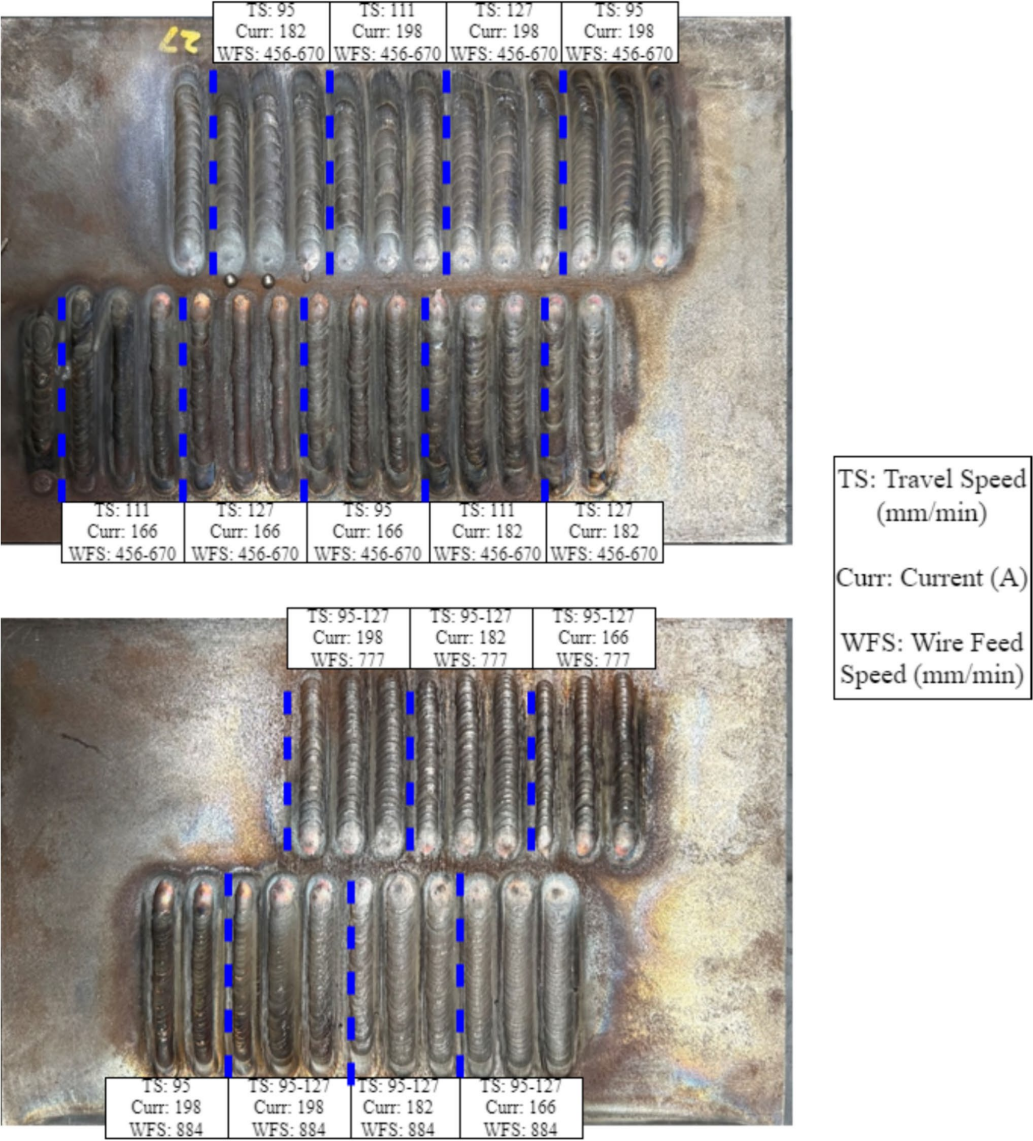
Bead-on-plate weld matrix used for process parameter tuning. Welds were deposited on S275 steel using varying heat inputs to evaluate bead shape and sidewall engagement

Initial trials consisted of bead-on-plate welding to identify process parameters that produced consistent weld morphology and favorable penetration characteristics. The goal was to obtain tall, uniform beads with minimal ripple effect, enabling reliable sidewall inspection and deliberate defect generation. A range of travel speeds and wire feed rates was explored, as shown in Table 1 , and visual inspection was used to identify stable operating conditions, as shown in Fig. 2. The optimized parameters were subsequently used both to induce artificial lack of sidewall fusion and to conduct in-process monitoring trials under consistent welding conditions. After establishing stable welding parameters, controlled defects were introduced to validate the sensitivity of the system offline and during welding. Programmed torch deviation was used to generate reproducible LOSWF flaws, enabling direct assessment of detection sensitivity and correlation between signal response and fusion integrity.

**Table 1:** Process parameters for bead-on-plate experimentation

Parameters	Units	Process parameters
Wire feed speed (WFS)	mm/min	[456, 563, 670, 777, 884]
Travel speed (TS)	mm/min	[95, 111, 127]
Current (Curr)	A	[166, 182, 198]

## Automated LOSWF generation

**Fig. 3:**
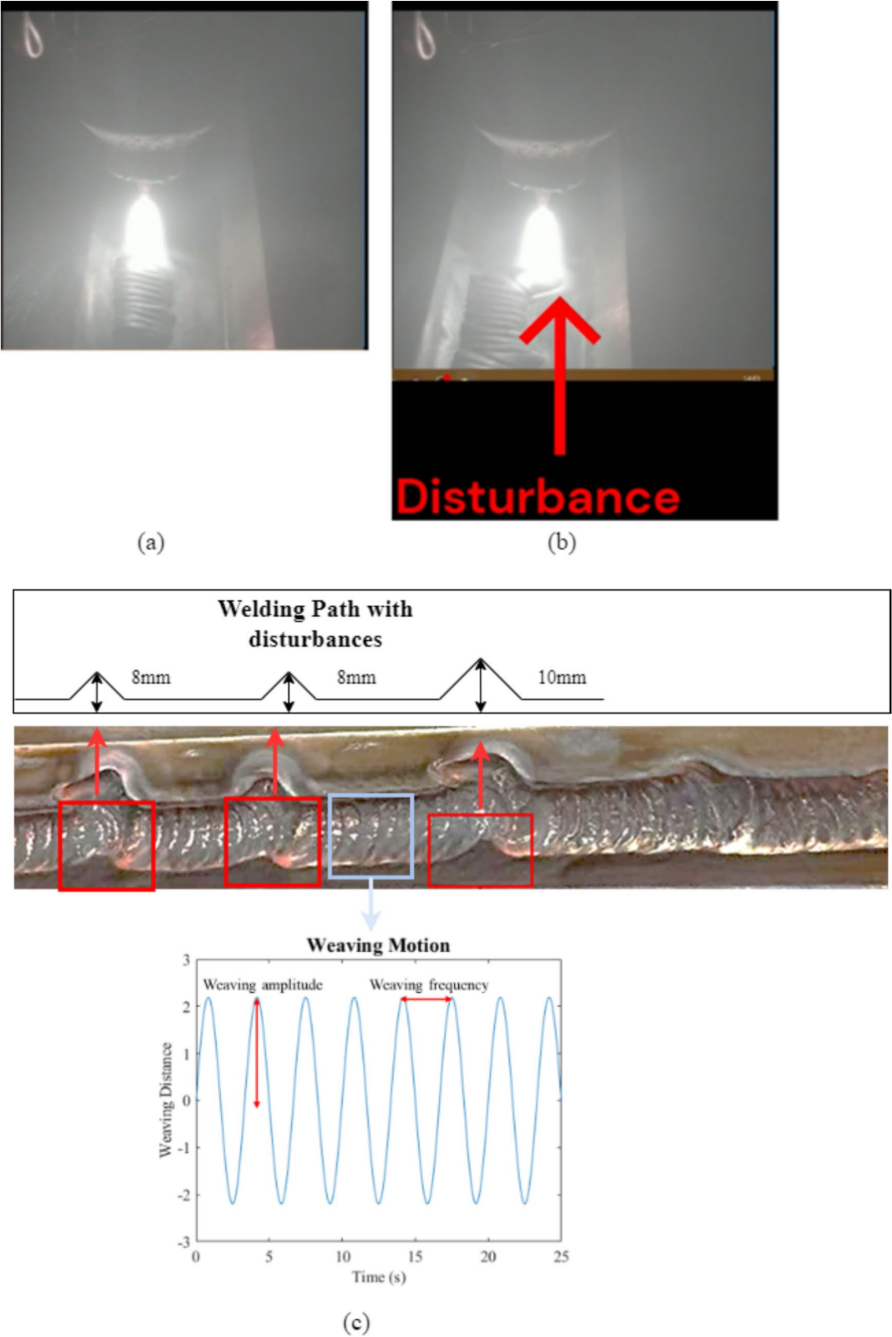
Illustration of welding disturbances and weaving motion during LOSWF generation. **a** Normal welding condition. **b** Welding under a disturbance showing a visible path deviation. **c** Welding path schematic with disturbances of 8 mm and 10 mm, corresponding to geometric deviations along the weld bead. The bottom plot shows the sinusoidal weaving motion with an amplitude of 2.2 mm and a frequency of 0.3 Hz, used for generating the LOSWF

To induce controlled lack of sidewall fusion (LOSWF) defects, the welding torch was deliberately shifted away from the bevel face during deposition, causing the arc to miss the joint sidewall while still forming a bead on the plate surface. This misalignment was executed in real time under visual monitoring, as shown in Fig. 3b, where the live weld pool visibly deviates from its nominal path, as shown in Fig. 3a. A sinusoidal weaving path was applied using a weaving frequency of 0.3 Hz and an amplitude of 2.2 mm, which determined the lateral oscillation rate and maximum deviation of the torch from the weld centerline. This motion facilitated uniform heat distribution and consistent bead formation across the joint width. The procedure was carried out under process conditions previously optimized for stable penetration, ensuring that the observed defects were exclusively due to geometric mispositioning rather than erratic process variables. Two disturbances of 8 mm and one of 10 mm were introduced to analyze the sensitivity, measuring the distance between the middle of the weaving motion and the bottom corner as shown in Fig. 3c.

## Automated LOSWF and post-process ultrasonic detection

**Fig. 4:**
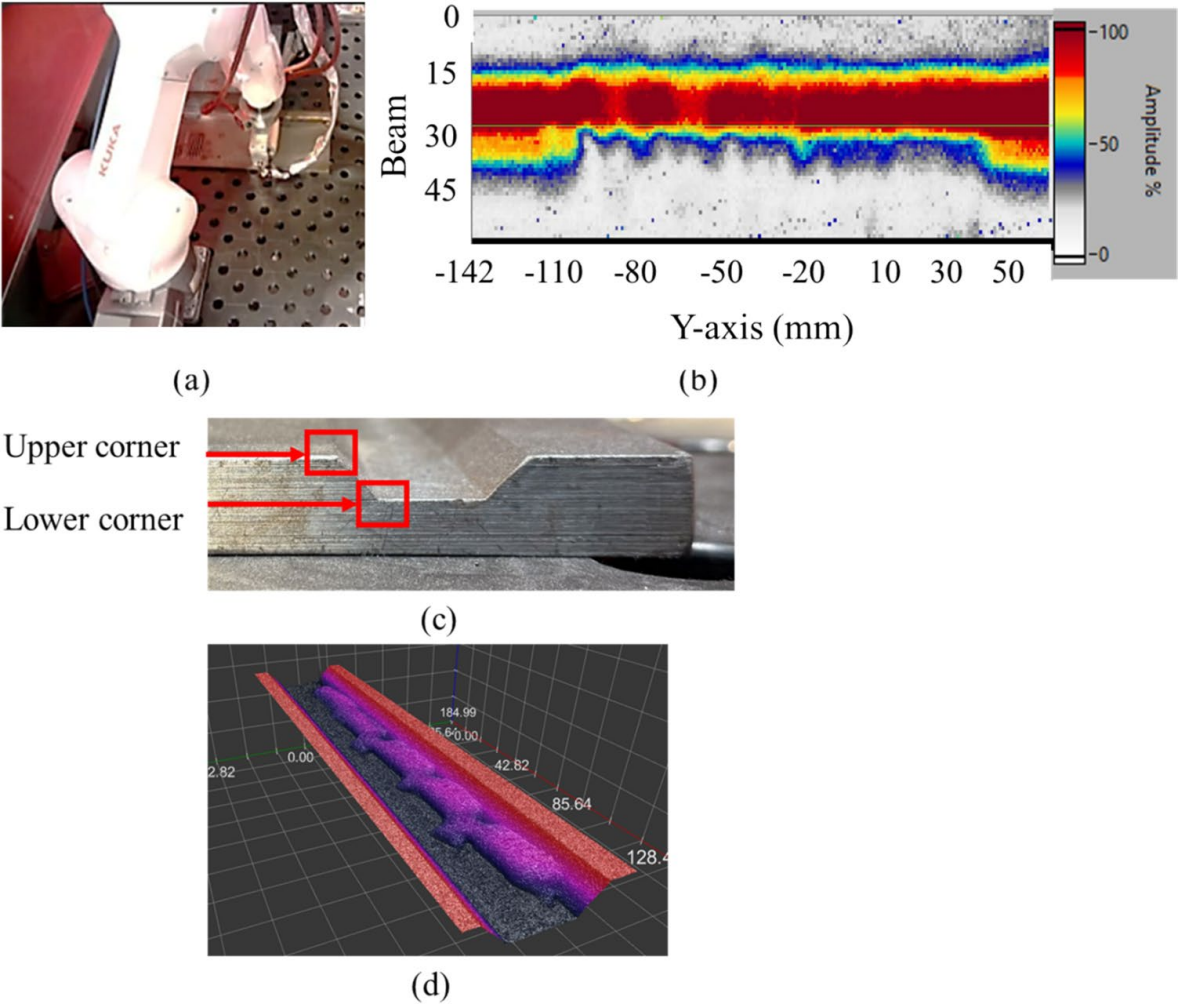
Post-weld robotic inspection using the roller-probe. **a** Live image during scanning. **b** Corresponding C-scan showing amplitude drop near weld centerline due to artificial sidewall fusion defect, **c** Bevel corner definition. **d** Point cloud reconstruction of sample

To verify the above LOSWF generation strategy, automated ultrasonic inspection was conducted after weld completion and at room temperature using a RollerProbe affixed to a 6-axis robotic arm. The RollerProbe was programmed to follow a single longitudinal path, offset by 40 mm from the plate edge to align the inspection line directly over the upper bevel corner—matching the location of the expected sidewall fusion defects, as shown in Fig. 4a. A constant force-torque feedback loop with 125 N of force ensured uniform coupling throughout the pass. Data was collected using B-Scans, enabling reconstruction of high-resolution C-scans. A gain of 35 dB, a subaperture of 8 elements, the spacing of a single element, and an inspection angle of 45° was used. Furthermore, a sampling rate of 100MHz, a voltage of 200 V, and a gate of 2000 to 4508 samples (40 to 90 μs) were used. As shown in Fig. 4b, zones corresponding to intentionally induced LOSWF defects showed measurable echo attenuation and backwall disruption, validating the probe’s sensitivity under static, room-temperature conditions. For cross-validation, the sample was then scanned using a Micro-Epsilon Scan-Control 2910-100/B to establish a ground truth for ultrasonic sensitivity. The minimum height was measured in a range of −12 to −4 mm, with a spatial resolution of 0.08 mm in the *Y* direction (welding direction), 0.03 mm in the *X* direction (bead width), and 0.01 mm in the *Z* direction (bead height) in order to establish the ground truth for disturbance depth relative to the base plate.

## Automated real-time LOSWF generation and ultrasonic detection

**Fig. 5:**
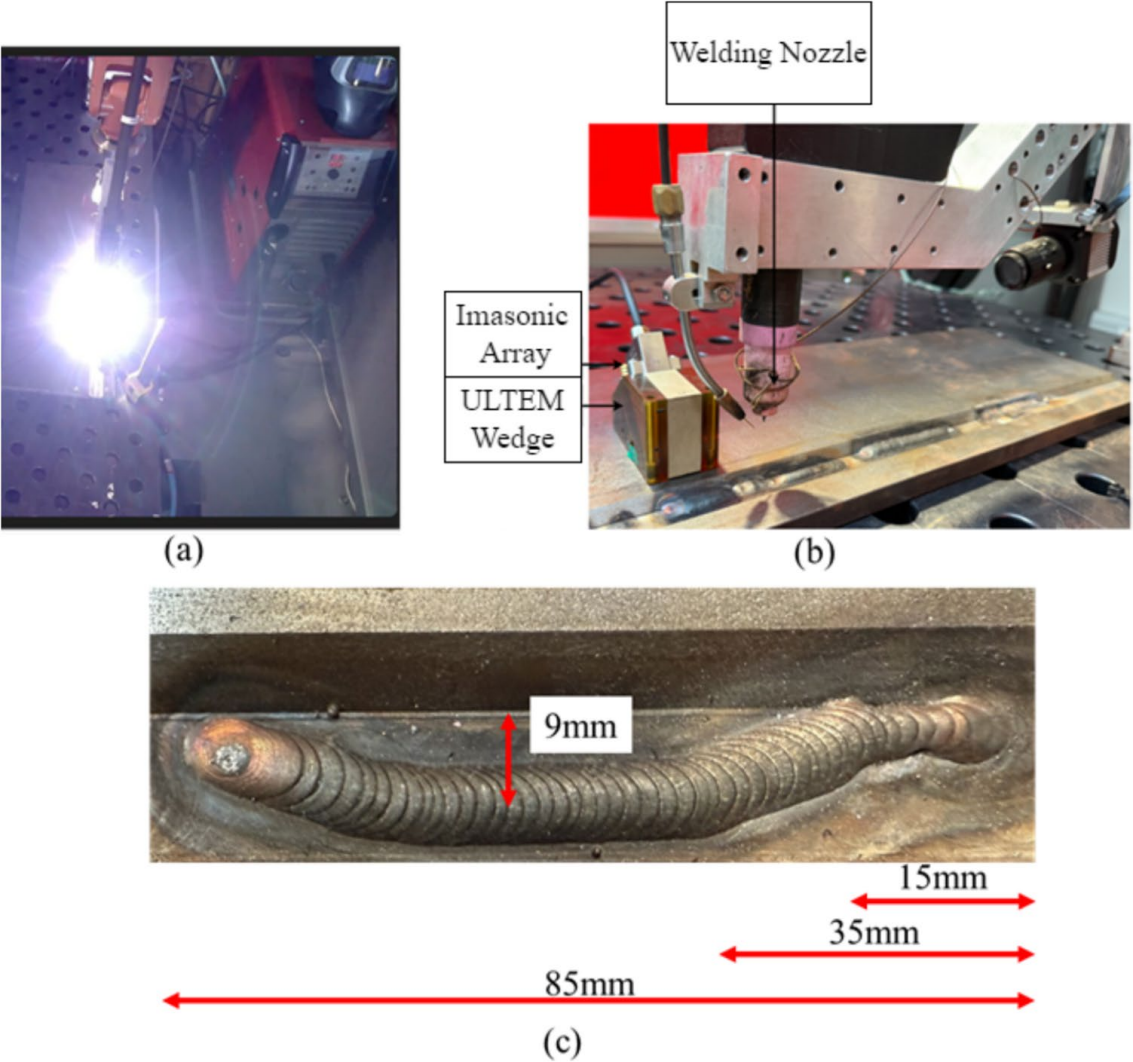
Composite view of in-process phased array experimental setup. **a** Robot-mounted TIG welding torch depositing a single bead during live ultrasonic acquisition. **b** Stationary ULTEM wedge housing a 5 MHz 64-element Imasonic probe positioned 20 mm from the weld bevel for signal acquisition during deposition, and **c** in-process experiment with defect generation

To then investigate the real-time ultrasonic detection of LOSWF during weld deposition, a single-pass weld was deposited while in-process ultrasonic inspection was simultaneously performed. A 5 MHz, 64-element phased array probe was used, coupled to a high-temperature wedge designed to transmit ultrasonic waves at a nominal refracted angle of 55° into the material. In order to monitor the lack of sidewall fusion, the 6-dB drop method was used on the C-Scan to determine the position of the upper and lower bevel corner. The wedge was positioned with the front face 20 mm from the upper bevel corner. A steering angle of 45°, with longitudinal transmission mode, was selected to monitor the melt pool with a gain of 35 dB, a subaperture of 8 elements, and the spacing of a single element. Furthermore, a sampling rate of 100MHz, a voltage of 200 V, a gate from 1800 to 2600 samples (36 to 52 μs), and no focusing were used. This configuration enabled real-time B-scan acquisition during welding (Fig. 5b). The torch followed a 50-mm linear path without any deliberate deviation, ensuring stable thermal and geometric conditions, as shown in Fig. 5a. The wedge was positioned in the middle of the bead, pointing to an index of 25 mm. Then, a trapezoidal disturbance was created, as shown in Fig. 5c, to simulate lack of sidewall fusion during welding. A distance of 5 mm was selected between the edge of the bead and the bottom corner. This test served as a baseline to assess signal quality, interference effects, and the feasibility of live defect detection during robotic welding.

## Automated real-time LOSWF ultrasonic detection

**Fig. 6:**
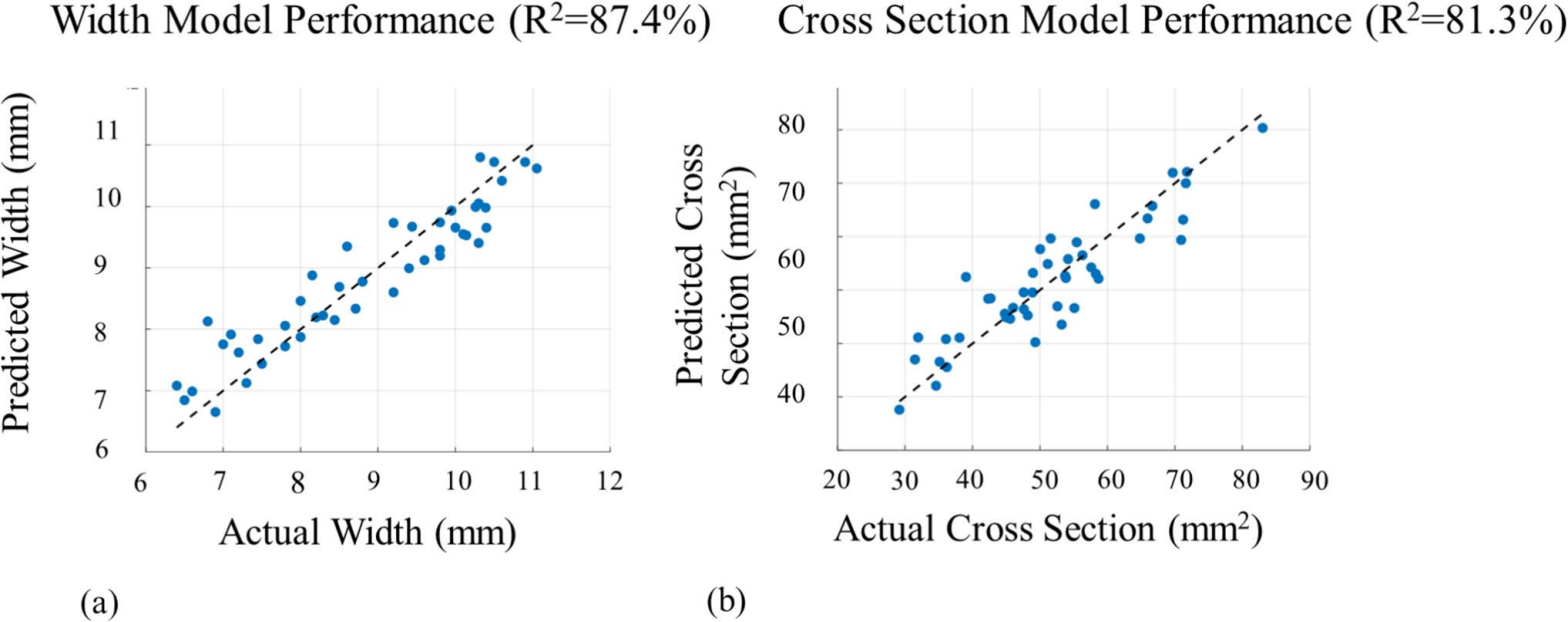
**a** Predicted versus actual weld bead width for bead-on-plate trials. **b** Predicted versus actual weld cross-sectional area for bead-on-plate trials

To ensure that the selected welding conditions produced predictable and reproducible bead morphology, linear lasso regression was performed on the bead-on-plate trials across a range of travel speeds and wire feed rates. It was selected to retain only the subset of features with the highest predictive capability, while shrinking the coefficient of parameter combinations with minimal impact. Figure 6 shows the relationship between actual and predicted values from the regression models of weld width and cross-sectional area. The width and height were manually measured using a caliper across 5 different points of the bead to calculate the average, and the cross-sectional area was calculated by considering the height and width and calculating the elliptical area defined by them. The points were selected in increments of 15 mm, to fully characterize dimensional deviation, starting 5 mm after the start of the weld. Strong linear trends were observed across the full range of experimental conditions (7–11 mm in height, 30–80 mm^2^ in cross-section), with strong statistical fits of 87.4% and 81.3% for width and cross section respectively, validating that weld geometry was a consistent function of input parameters on the deployed system. These results confirm that bead dimensions—particularly sidewall engagement—could be systematically manipulated and reproduced during both flaw induction and in-process inspection trials. The optimal selected parameters are 198 A, 95 mm/min, and a wire feed speed of 884 mm/min for a bead height of 2.4 mm and a width of 11.05 mm. These parameters were selected based on visual evaluation of bead morphology to maintain process consistency and achieve an appropriate bead height, ensuring a detectable resolution above 2.0 mm for ultrasonic inspection and ensuring sufficient bevel wetting.

**Fig. 7:**
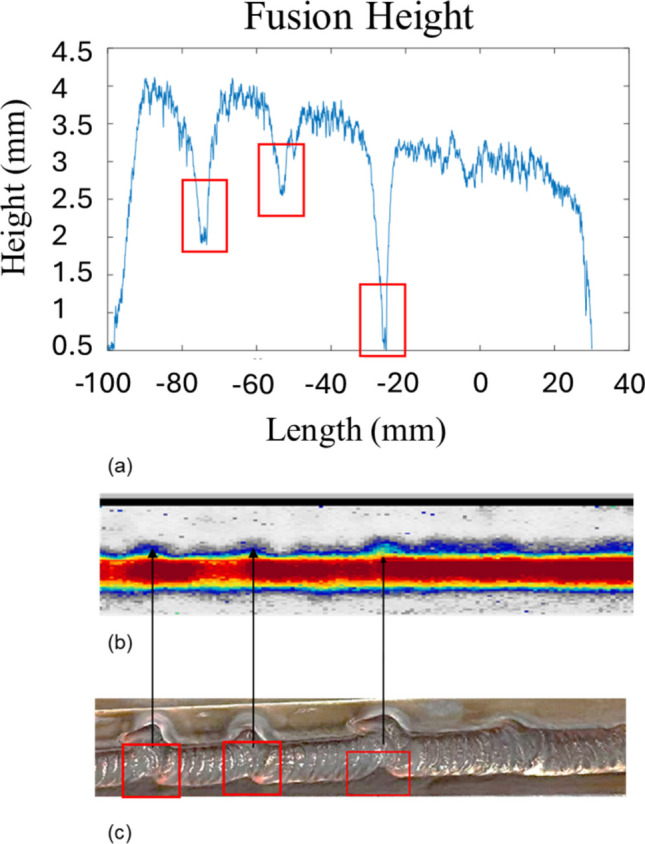
Composite defect validation from the disturbance experiment. **a** Extracted bevel length trace from laser scan, showing changes in height corresponding to disturbance. **b** Corresponding C-scan amplitude map from phased array data showing signal attenuation near the weld centerline, aligned with the defect zone. **c** Surface macrograph with a visible fusion defect (highlighted in the red box)

To assess the detectability of artificial LOSWF defects, deliberate torch deviation was employed to locally shift the arc away from the fusion face during deposition. As shown in the image (Fig. 7c), the resulting bead exhibited visible underfill and inconsistent wetting near the sidewall, confirming successful defect induction. A corresponding plot of bevel length across the weld (Fig. 7a) shows a clear negative spike in profile deviation at the same Y-position, verifying that the defect was also geometrically distinct. Depending on the lateral offset, the disturbance depth changes from 2 to 4 mm, with the same disturbance of 8 mm producing differing depths due to proximity and the effect of thermal gradient. Critically, phased array C-scan data (Fig. 7b) captured a pronounced increase in echo size at this location, with echo intensity markedly increased in the affected region. While smaller disturbances were less clearly distinguishable due to background variation and bead irregularity, the most severe defect produced a detectable ultrasonic signature aligned with visual and geometric cues. These results confirm that phased array ultrasonics can reliably detect sidewall fusion defects of sufficient size, highlighting the value of geometric-acoustic correlation in weld monitoring and inspection.

**Fig. 8:**
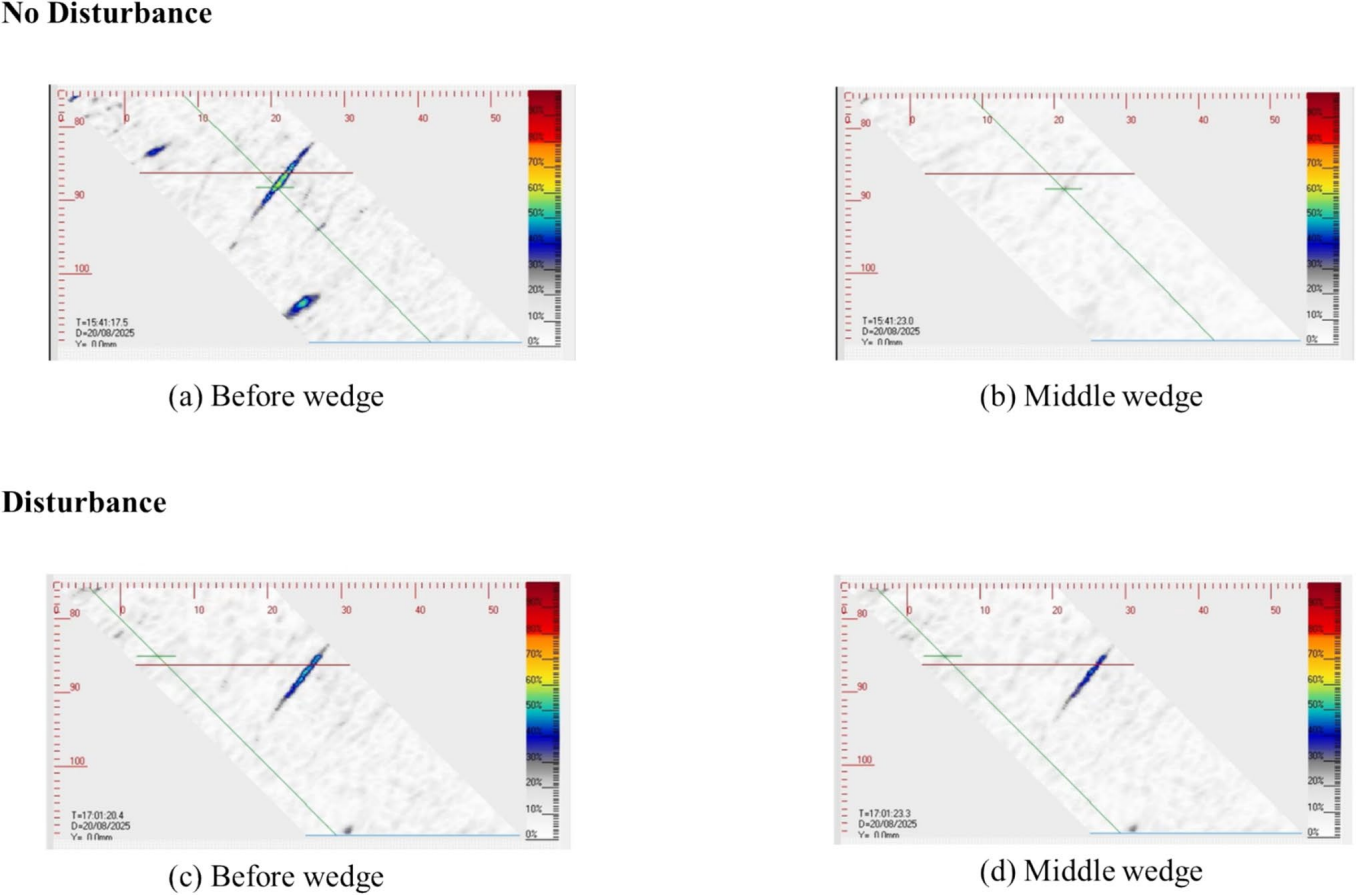
B-scan sequence during in-process welding with a static wedge for no disturbance: **a** clear signal before arc arrival; **b** signal loss as arc passes probe, and for disturbance: **c** clear signal before arc arrival and **d** signal stability as arc passes probe

To evaluate the feasibility of real-time ultrasonic feedback during deposition, a static wedge configuration was employed with the phased array probe positioned ahead of the weld torch path. While artificial lack-of-sidewall-fusion (LOSWF) defects were introduced under controlled bead-on-plate conditions, their acoustic and geometric responses closely mimic those reported in real-world fusion flaws. Artificial LOSWF defects are widely used in weld qualification and ultrasonic validation studies as reliable surrogates for service-induced flaws, since their geometric and acoustic responses closely replicate those of real defects [27–29]. Nonetheless, extrapolating to complex multi-pass welds requires caution, as thermal gradients, constraint effects, and process variability may alter defect signatures. These experiments therefore establish a foundational validation framework, with ongoing work aimed at extending applicability to production-scale welding scenarios. B-scan images acquired during single-pass GTAW welding revealed distinct signal behaviour in two stages for both experimental scenarios. For the lack of disturbance, before the arc arrived, as shown in Fig. 8a, we observed clear echoes from the bevel sidewall, indicating strong coupling and material response. Smaller echoes surrounding the main indication correspond to scattered or mode-converted reflections at the bevel interface, confirming proper beam engagement during skip inspection. However, when the arc passed directly in front of the wedge, as shown in Fig. 8b, the ultrasonic longitudinal signals were absorbed into the molten pool. In contrast, when the experiment is repeated, as shown in Fig. 8c, but including an artificial lack of sidewall fusion disturbance directly in front of the wedge, the signal is still apparent when the torch approaches the middle of the wedge as the wave does not enter the molten pool, as shown in Fig. 8d. Understanding this behavior is essential for developing closed-loop welding inspection systems that must interpret ultrasonic data in near real-time during material deposition. Notably, the temporary signal loss during proper fusion—caused by phase mixing—provides a reliable indicator of bonding integrity. Experimental results demonstrate that in-process fusion face monitoring is feasible and presents a binary detection problem: the ultrasonic longitudinal wave is absorbed by the molten pool in fused joints, while it is not in lack of fusion cases. This finding opens a promising pathway for real-time, feedback-driven welding quality control.

## Conclusion

This work demonstrates a novel approach for real-time detection of lack of sidewall fusion (LOSWF) using phased array ultrasonics integrated with automated welding systems. By deploying phased array ultrasonics during the welding process, we achieved real-time B-scan monitoring which revealed characteristic signal dropout and recovery patterns correlating with fusion quality. This transient behaviour from 60% normalized amplitude to 0% for proper fusion provides an immediate proxy for sidewall fusion status during active welding. An important limitation of this work is the difficulty in extracting quantitative information from the fusion face during welding, which constrains the design of more advanced control algorithms. As observed in Fig. 7, thermal gradients can further narrow the monitoring window due to signal degradation. Additionally, in complex multi-pass welds, elevated interpass temperatures, variable geometries, and arc-induced noise may degrade signal stability and introduce ambiguity in distinguishing genuine fusion loss from transient acoustic fluctuations. To enable robust fusion-face control near the melt pool, integration with complementary sensing—such as visual feedback or melt pool width monitoring as demonstrated in prior work [21], as well as positional feedback and thermal compensation—will be essential.

Controlled experiments with artificially induced LOSWF defects validated detection sensitivity and established baseline characterization metrics, while thermal disturbance trials quantified signal behaviour under realistic welding conditions. The results demonstrate that ultrasonic signals remain interpretable, enabling rapid evaluation of fusion integrity at the point of deposition using signal amplitude as the main criteria.

These findings enable direct integration of ultrasonic imaging into intelligent welding manufacturing systems, supporting both predictive defect identification and real-time quality confirmation. This embedded NDT approach addresses key Industry 4.0 requirements for autonomous quality assurance, reducing post-process inspection burden while enhancing manufacturing reliability in safety-critical applications.

Future work will focus on fully autonomous tandem robotic systems combining real-time welding with trailing PAUT inspection. Key challenges include maintaining signal integrity under challenging interference and developing adaptive control algorithms that respond to ultrasonic feedback. This represents a critical step toward fully closed-loop intelligent welding systems where quality monitoring drives real-time process adaptation, establishing the foundation for next-generation automated manufacturing platforms.

## Data Availability

Not applicable.
